# The Effects of Abdominal Hypopressive Training on Postural Control and Deep Trunk Muscle Activation: A Randomized Controlled Trial

**DOI:** 10.3390/ijerph18052741

**Published:** 2021-03-08

**Authors:** María del Mar Moreno-Muñoz, Fidel Hita-Contreras, María Dolores Estudillo-Martínez, Agustín Aibar-Almazán, Yolanda Castellote-Caballero, Marco Bergamin, Stefano Gobbo, David Cruz-Díaz

**Affiliations:** 1 Department of Health Sciences, Faculty of Health Sciences, University of Jaén, E-23071 Jaén, Spain; marmoreno92@gmail.com (M.d.M.M.-M.); fhita@ujaen.es (F.H.-C.); aaibar@ujaen.es (A.A.-A.); dcruz@ujaen.es (D.C.-D.); 2 Department of Statistics, Faculty of Experimental Sciences, University of Jaén, E-23071 Jaén, Spain; mdestudi@ujaen.es; 3 Department of Medicine, University of Padova Palazzina ex SemeioticaMedica-Via Ospedale Civile, 105 35128 Padova, Italy; marco.bergamin@unipd.it (M.B.); stefano.gobbo@unipd.it (S.G.)

**Keywords:** postural control, balance, deep trunk muscles, abdominal hypopressive training

## Abstract

Background: Abdominal Hypopressive Training (AHT) provides postural improvement, and enhances deep trunk muscle activation. However, until recently, there was a lack of scientific literature supporting these statements. The major purpose of this study was to investigate the effect of AHT on posture control and deep trunk muscle function. Methods: 125 female participants aged 18–60 were randomly allocated to the Experimental Group (EG), consisting of two sessions of 30 min per week for 8 weeks of AHT, or the Control Group (CG), who did not receive any treatment. Postural control was measured with a stabilometric platform to assess the static balance and the activation of deep trunk muscles (specifically the Transverse Abdominal muscle (TrA)), which was measured by real-time ultrasound imaging. Results: The groups were homogeneous at baseline. Statistical differences were identified between both groups after intervention in the Surface of the Center of Pressure (CoP) Open-Eyes (S-OE) (*p* = 0.001, Cohen’s d = 0.60) and the Velocity of CoP under both conditions; Open-Eyes (V-OE) (*p* = 0.001, Cohen´s d = 0.63) and Close-Eyes (V-CE) (*p* = 0.016, Cohen´s d = 0.016), with the EG achieving substantial improvements. Likewise, there were statistically significant differences between measurements over time for the EG on S-OE (*p* < 0.001, Cohen´s d = 0.99); V-OE (*p* = 0.038, Cohen´s d = 0.27); V-CE (*p* = 0.006, Cohen´s d = 0.39), anteroposterior movements of CoP with Open-Eyes (RMSY-OE) (*p* = 0.038, Cohen´s d = 0.60) and activity of TrA under contraction conditions (*p* < 0.001, Cohen´s d = 0.53). Conclusions: The application of eight weeks of AHT leads to positive outcomes in posture control, as well as an improvement in the deep trunk muscle contraction in the female population.

## 1. Introduction

Postural control is defined as a motor skill that maintains the body´s vertical axis within the ends of the support base [1] in any static or dynamic condition, so it is a prerequisite for dexterous coordination and an integral component of overall motor skill [1,2]. The performance of postural stability is integrated by mechanisms that determine the processes of balance control, provided by the complex activity of the control system, which includes neuromuscular synergies and the sensory and cognitive systems of anticipative and adaptive mechanisms [3,4,5]. Previous research has shown that co-activation of the spinal stabilizer muscles, including systems like the thoracic diaphragm, deep trunk muscles (especially the transverse abdominal (TrA), and pelvic floor muscles (PFM)), have a major role in postural stability [6,7]. The correct activation of the deep trunk muscles results in a significant improvement of the lumbar spine stability through greater intra-abdominal pressure and increased stress of the thoracolumbar fascia (by direct connection with the TrA) [6]. In healthy individuals, TrA activity is usually triggered before any postural disturbance, to protect the lumbar region from imposed forces. [8]. If this perturbation is not anticipated, balance disruption may occur [9]. As such, understanding how abdominal muscles respond specifically in postural control is a key element for limiting the risk of injuries, as the center of the movement chain is located in the trunk [10]. 

Evidence suggests that physical training focused on deep trunk muscle stabilization improves postural control [1]. Caufriez et al., in 1980 [11], proposed Abdominal Hypopressive Training (AHT) as a method to prevent pelvic floor dysfunction after pregnancy [12], then as training to improve postural control [13]. It consists of postural and breathing techniques [14,15] which decrease the pressure in three compartments: the thoracic, abdominal, and perineal [11]. It has been hypothesized that complete exhalation, followed by apnea, blocks the glottis and opens the chest cavity in such a way that the diaphragm is stretched, causing involuntary trunk deep muscle activation [12,13,14]. This maneuver, added to postural techniques, aims to induce activation of tonic muscle fibers (type I), which should increase the synergistic activation of all postural muscles, including the deep trunk muscles [13,14,16]. Thus, this training reinforces the abdominal binder and stabilizes the lumbar spine. Benefits such as improved flexibility of the lumbar and hamstring muscles, and restructuring of the posture, have also been suggested [11,16,17,18]. Regarding postural techniques, AHT is performed in a sequence of positions, which usually start standing up and end in a supine position, passing through kneeling, quadruped, and sitting positions [13].

This study aimed to assess the effects of AHT on posture control and deep trunk muscle function, focusing on the activity of the TrA.

## 2. Materials and Methods

### 2.1. Study Design and Subjects

A randomized controlled trial was performed. Participants were recruited by advertisements in public institution (Hospitals and University) and social media (Instagram and Facebook). Inclusion criteria included female gender and aged 18 to 60; individuals were excluded if they presented with contraindications to AHT participation (cardio-respiratory disease, untreated arterial hypertension, pregnancy, hiatus hernia) [13], had participated in AHT in the past 2 years, had balance or neurological deficits, experienced vestibular alterations, or had an autoimmune disorder. Women who were not able to commit to or did not attend three sessions of this study were also excluded. 

The principal investigator scheduled meetings with people interested in the project. All women, who decided to participate signed an informed consent from before the beginning of the study. The participants who met the inclusion criteria were randomly assigned to the Experimental (EG) or Control Group (CG). This allocation was conducted by the OxMaR [19] system at a 1:1 ratio. The participants knew their group before starting the intervention; this was communicated through sealed opaque envelopes created by an independent researcher not otherwise involved with the study.

The present study had the approval of the Human Ethics Committee of the University of Jaén (NCT04343599), and complied with the statement CONSORT guidelines [20].

### 2.2. Procedure

**Figure 1:**
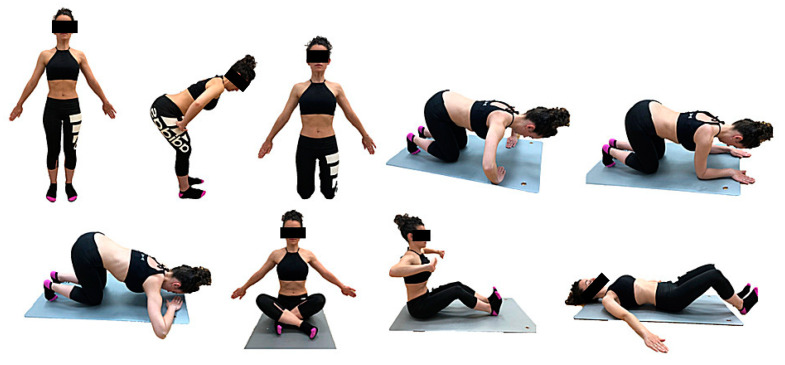
Progression of hypopressive postures.

**Figure 2:**
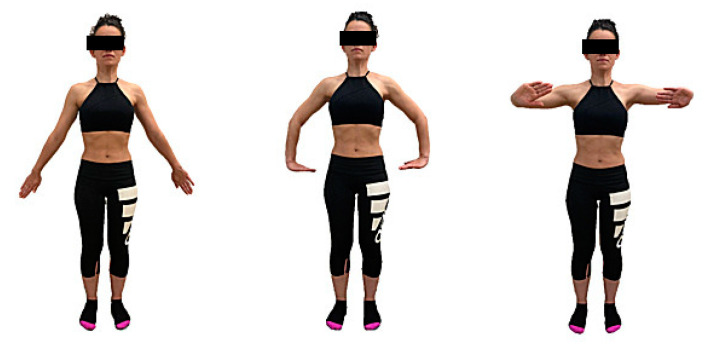
MMSS variant in hypopressive positions. (1) Extension and pronation. (2) Internal rotation shoulder, 90º flexion elbow and wrist, hand push to the floor. (3) Internal rotation and 90º flesion shoulder, 90º flexion elbow and wrist, push forward.

Participants allocated to the intervention group received an intervention of AHT, based on the Low-Pressure Fitness (LPF) protocol, of two sessions of 30 min per week for 8 weeks [21]. The first two sessions consisted of learning breathing techniques and postural fundamentals of AHT (Table 1), which are maintained in all postures and their variants in the upper limbs (Figure 1 and Figure 2). In the rest of the sessions, the complete progression of hypopressive postures (from standing to supine) was performed. The participants preserved an attitude of constant muscular activation. A hypopressive maneuver (expiratory apnea) was executed for every hypopressive posture, after three breath cycles. Three repetitions were performed with each posture, and the patients changed their posture during the expiration maneuver. The rhythm of breathing and exercises was guided by the instructor, who supervised the correct execution. 

The participants of the CG engaged in no treatment during the study. Telephone contact was maintained to ensure that the women who did not receive treatment were not engaging in any other specific deep trunk muscle physical training in any activity that they were not performing previously, since this could have interfered with the results of this study.

### 2.3. Outcome Measures, Variables, and Instruments

The primary outcome measure was postural control, assessed with a stabilometric platform, with TrA activity measured by real-time ultrasound imaging included as the secondary outcome measure. Both groups were assessed by an independent assessor at baseline and after the intervention (8 weeks later). Sociodemographic and clinical data at baseline can be found in Table 2.

### 2.4. Primary Outcome

Postural control was the primary outcome measure. This continuous variable was assessed by a stabilometric platform (Sensor Medica, Rome, Italy), with an active surface of 400 × 400 mm and a frequency of 30 Hz. The Free-Step Standard 3.0 software (Sensor Medica, Rome, Italy) which has been employed in previous studies [22,23] was used to calculate the center of pressure (CoP) movement. Postural control assessment was performed following the same methodology of previous studies and based on Romberg test [24,25]. This test provided stabilometric measures under two conditions in succession: an Open (OE) and a Closed Eyes (CE). Each measure lasted 30 s and there was a 1-min interval between tests [26]. Participants on the platform were kept immobile, assuming a comfortable posture with the arms beside the body, legs parallel, and bare feet at a 30º angle. The stabilometric measures analyzed in this study were participant´s CoP amplitude in the mediolateral (RMSX) and anteroposterior (RMSY) orientation (mm), the area coated (S, mm^2^), and the velocity movement of CoP (V, mm/s). Moderate to substantial reliability has been demonstrated for this pressure platform in terms of the stabilometric variables reviewed in this trial, with an intraclass correlation coefficient of 0.495 to 0.832 [23,27].

### 2.5. Secondary Outcome

TrA activation was the secondary outcome (continuous variables). A SonoScape S2 ultrasound system was used with an 8 MHz linear transducer. The measurement process followed previous research [28]. The position of the participants was supine, with arms along the body, pelvis in a neutral position (equal distance between anterior and posterior superior iliac spines), knee flexion of 45°, and feet supported on the stretcher. The transductor was placed on the right side of the participant, drawing a line between the last rib and the iliac crest, and about 2.5 cm distal of the middle point was the thickness of the TrA. The measure was taken vertically at 1 cm between the edge of the muscle and the interface of the internal oblique muscle (IO) origin. Three repeated values were taken at rest and in contraction conditions (mm). Each image was captured by keeping the transducer in the same position, and with the same pressure. The resting measurement was taken during exhalation during comfortable breathing, and the contracting measurement was taken at the moment of maximum expiration; the participants were instructed to bring the navel towards the stretcher and towards the head, without losing the neutral pelvic position [29]. An assessor instructed the women on how to do this and control the pelvis, thus achieving the most selective contraction of the TrA.

### 2.6. Statistical Analysis 

The sample size was calculated using Ene 3.0 (GlaxoSmithKline, SA, Madrid, Spain) [27,30,31], based on stabilometric paramenter included in previous studies [32], to obtain a statistically significant difference using stabilometric scores as the dependent variable, with a power of 0.80, a significance level of 95%, and considering an estimated drop out of 15%, 49 participants per group were required. SPSS statistical software, version 21.0 (SPSS, Inc., Chicago, IL, USA), was employed for the statistical analyses. Data were calculated as frequencies (percentages) and mean ± standard deviation for categorical and continuous variables respectively. We used the Kolmogorov–Smirnov test to check the normal distribution of all continuous variables. The Student´s t-test and the Chi-square test (Fisher’s exact test when the number of cases was small) was used to determine possible differences between continuous and categorical variables respectively. An ANOVA with a 2 × 2 design was applied, where the between-group component was determined by the intervention (EG and CG), and the within-group component was the time of measurement (pre and post-intervention). Results were considered statistically significant at a *p*-value < 0.05. Eta-squared was used for the effect size of the main effects. Cohen´s d was used to calculate effect sizes of the specific group × time interactions, which were considered as small (Cohen´s d = 0.2), medium (Cohen´s d = 0.5), and large (Cohen´s d = 0.8) [33].

## 3. Results

**Figure 3:**
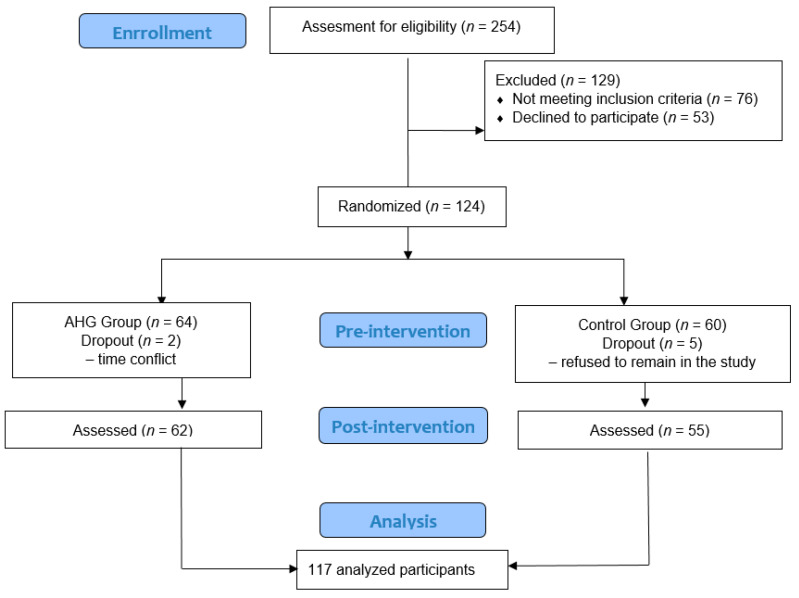
CONSORT Flowchart of patient selection and allocation.

From the initial sample of 254 participants, 76 did not meet the inclusion criteria and 53 declined to participate. A total of 125 women were allocated to each group (64 to EG and 60 to CG), and 117 participants completed the study. Seven women did not complete the study; five participants in the CG did not complete the last assessment, and the other two women from the EG ceased participation due to lack of availability (Figure 3). There were no differences at baseline in the studied variables and sociodemographic data (Table 2). The mean ± SD age of the participants was 45.65 ± 8.86 years; the mean ± SD values for weight and height were 63.59 ± 10.59 and 162.56 ± 5.95, respectively, and the mean BMI ± SD was 24.03 ± 3.63. The percentage of smoking women was 36.8% in the EG and 63.2% in the CG. A total of 29 and 34 participants reported exercising in the EG and CG, respectively. 

### 3.1. Postural Control

The results of the EG post-intervention showed an improvement of all variables related to postural control, however not all were statistically significant (Table 3).

**Figure 4:**
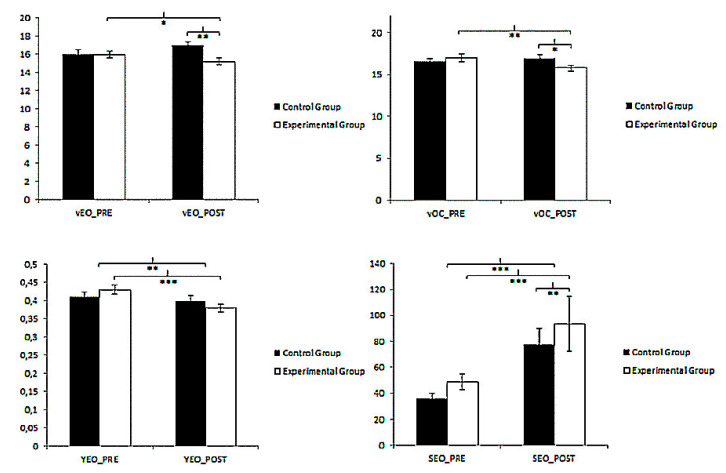
Between-group and within-group comparison of VOE, VCE, YOE, and SOE scores. VEO: mean speed of displacement of the pressure center with open eyes; VOC: mean speed of displacement of the pressure center with eyes closed; YEO: mean value of anteroposterior oscillations of the pressure center with open eyes; SEO: surface with open eyes. * *p* < 0.05; ** *p* < 0.01; *** *p* < 0.001.

The Romberg test under closed-eyes only showed statistically significant differences in the V-CE values concerning the Group × Time interaction: F(1.117) = 7.648, *p* = 0.0070 η^2^ = 0.61. An exhaustive analysis yielded statistically significant changes between groups after the intervention: t(117) = 2.438; *p* = 0.016, with a medium effect size (Cohen´s d= 0.45). The measures at pre and post-intervention found significantly higher values for the EG: t(62) = 2.843; *p* = 0.006 (Cohen´s d = 0.39). The rest of the stabilometric variables were not statistically significant (Figure 4).

Under the open-eyes condition, statistically significant differences were observed in all outcomes measures except RMSX-OE. Concerning RMSY-OE, a detailed analysis found statistically significant changes between pre and post-treatment measures in women who received AHG; t(62) = 4.719; *p* < 0.001 (Cohen´s d = 0.54). In V-OE and S-OE, the results showed significant differences between pre and post-measurement in the EG: t(62) = 2.116; *p* = 0.038; Cohen´s d = 0.27 and t(62) = 5.517; *p* < 0.001; d = 0.99; respectively. Significant differences were also found between both groups at post-intervention in both stabilometric variables; V-OE: t(117) = 3.434; *p* = 0.001; d = 0.63 and S-OE: t(117) = 3.377; *p* = 0.001 d = 0.60. 

### 3.2. TrA Activation

In both conditions (muscle contraction and relaxation), the women of the EG showed higher values than the women of the other group. However, only under contraction were the Time (F(1.117) = 15.44, *p* < 0.001, η^2^ = 0.117) and the Group × Time (F(1.117) = 13.057, *p* < 0.001, η^2^ = 0.100) interactions statistically significantly different. A detailed analysis revealed statistically significant changes only over time of intervention measurements for the group receiving AHT (t(62) = −5.240, *p* < 0.001; d = 0.53) (Table 3).

## 4. Discussion

The present study was the first randomized controlled trial addressing the effects of AHT on postural control, measured by empirical variables. The obtained results suggest that postural stability and deep trunk muscle activation significantly improved after eight weeks of AHT in the female population. 

### 4.1. Postural Control

Stabilometric analysis showed positive effects on the surface of the area, the anteroposterior displacements, and the velocity of the CoP movement variables in the EG after AHT intervention. Previous studies [17,18] hypothesized the influence of AHT on postural variables; however, the present study employed an objective and validated instrument supporting this. Caufriez et al. [17] suggested that there was an improvement in the mobility of the pelvic and lumbar spine after AHT intervention. In line with the aforementioned manuscript, Rial et al. [18] observed an increase in the mobility of the spine in female football players after six weeks of AHT. Both studies used subjective assessment of postural variables, like biometric parameters, which could have an error coefficient of up to 15% [17]. Regarding postural assessments, the velocity of the CoP is an important fall predictor [27], and can be considered a valid and reliable outcome measure to determine postural improvements in combination with the provided information of the surface data, obtained with the stabilometric platform [33,34].

The results obtained in this study concerning postural control improvement are in agreement with the previous findings of Caufriez et al. [17], who hypothesized that hypopressive breathing and postures could increase the synergistic activation of the postural muscles [15]. This co-activation could be related to the feedforward strategies and automatic adjustments around balance [35]. Maintaining an ankle dorsiflexion position during AHT may lead to a significant improvement in the AP sway range after the intervention. This stimulus in the anteroposterior axis during AHT implies a change in the center of gravity, and challenges the maintenance of stability. This may lead to the improvement in the stabilization strategies on some systems, such as the ankle joint, which play an important role in static and dynamic balance [36].

Surface data obtained with the stabilometric platform showed within and between groups improvement in the EG. Information provided in the RMSY analysis has been deemed to be clinically relevant to postural assessment [4]. Participants allocated to the AHT intervention presented slight improvements in frontal sway range after the intervention. In contrast with the anteroposterior axis, where the ankle joint is dominant, the mediolateral has a predominance of the hip joint [2,37]. The positive findings of this study could be explained by the activation of the external rotators and hip adductors, which are affected by the posture hypopressive [15]. Another important finding in this study was the observed statistically significant differences under OE or CE conditions. It has been suggested that an increase in postural sway could be a compensatory mechanism employed by an individual when vision is restricted. An improvement in these parameters is seen with improved proprioception. Feedforward strategies are influenced by all afferent information arising from the somatosensorial system, including proprioception. Therefore, when vision is limited, the rest of the system needs to be compensated by the rest of the afferent elements [38,39].

### 4.2. Deep Trunk Muscles

Our results agree with previous studies reporting improvement in deep trunk muscles after AHT [15,16,40]. Navarro Brazález et al. [15] reported that deep abdominal muscles are activated between 25.4% and 35.3% of the rest condition performance of the supine hypopressive posture, and at 22.8% during the orthostatic hypopressive posture. We observed that there was an improvement of TrA during voluntary contraction but not at rest. These findings are consistent with a previous study, where it was suggested that hypertrophy is not possible after AHT because of the load characteristics and intensity during the described exercises [41]. Nevertheless, a longer period than eight weeks could lead to some improvement in this parameter, as suggested by some authors [41].

The transversus abdomini, which is made up of 75% type I muscular fibers vs. 4% type IIb, can be considered one of the principal deep trunk muscles involved in intraabdominal pressure regulation [42]. Taking into account the anatomic and physiological characteristics of this muscle group, the aim of this intervention was not focused on hypertrophy, but the proper activation timing of the TrA. The activation of this muscle group may play an important role in the improvement of some pathologies, such as in the lower back [43]. Previous research has suggested that the pre-activation of the trunk stabilizer muscles may contribute to better muscle activation of the rest of the muscles involved in movement during functional tasks [41]. The performance of apnea, coupled with the opening of the rib cage during hypopressive breathing, might be responsible for this improvement [15]. This maneuver attempts to reduce intra-abdominal pressure (IAP) and might result in synergistic contraction of the TrA, due to the important role of this muscle in the regulation of IAP [44]. Neural adaptation and greater central drive could be a plausible explanation for improvement during the contraction.

The findings regarding the improvement in the TrA activation are consistent with previous research [15,16,18,45]. Nevertheless, one of the strong points of this research is the ultrasound measurement of this parameter. The assessment of this muscle group is difficult due to the proximity of the muscles surrounding it. Therefore, the use of real-time ultrasound measurement is recommended over other assessment options, such as electromyography [28,46]. The risk of using surface electrodes is an overflow of other abdominal muscle contractions, known as “cross-talk” [47]. Nevertheless, the results obtained by ultrasound assessment and electromyography are generally similar [15,16,18,40].

### 4.3. Study Limitations

This study also had some limitations. Participants could not be blinded given the nature of the intervention and the control group did not receive a sham or placebo intervention. Furthermore, a longer follow-up period would be beneficial to determine the effects of AHT overtime

## 5. Conclusions

The results suggest that a program of eight-weeks of AHT (with twice-weekly sessions) has a beneficial role in the improvement of postural control parameters and transversus abdomini activation (as assessed with stabilometric platform and ultrasound respectively), in the female population. With respect to the stabilometric parameters, our findings showed an improvement in the velocity of the CoP movement with closed eyes and the surface of the area, as well as in the anteroposterior displacements, and the velocity of the CoP movement under open eyes condition. Thus, since, static balance is related to the functional status, it may contribute to better performance during task execution in which vision is involved. 

Furthermore, the importance of pelvic stabilization and coordination exercises in patients with some pathologies, such as lower back pain, has been widely addressed [1,6,28,48]. Analyzing the obtained results, AHT could be a complementary and additional intervention option for the improvement of deep trunk muscle activation. The decrease in intra-abdominal pressure during this exercise in contrast with other interventions could be considered an additional benefit in some population groups, such as women during the postpartum period, or those with any pelvic floor dysfuction [44]. 

## Figures and Tables

**Table 1:** Technical foundations of Abdominal Hypopressive Training (AHT)^14^.

Technical Foundations	Definition
**Autoelongation**	Axial stretching of the spine, tensioning of deep spine and back extensors
**Double chin**	Pulling the crown to the ceiling
**Decoaptation of the glenohumeral joint**	Scapula abduction and serratus activation
**Neutral pelvis**	Equal distance between anterior and posterior superior iliac spines
**Dorsal ankle flexion**	Parallel lower extremities with hip-width, slight knee flexion, and dorsal ankle flexion
**Gravity shaft overrun**	Imbalance of the anteroposterior axis involving variation of the center of gravity
**Diaphragmatic breathing**	Nasal inspiration with rib opening Braked mouth breathing
**Expiratory apnea**	Total exhalation Apnea maintained with a costal opening: closure of the glottis, voluntary contraction of the serratus majors and muscles of the upper airway, intercostals, scalenes, and sternocleidomastoid Diaphragm relaxation and cavity pressure reduction

**Table 2:** Baseline characteristics of the study group.

Demographic Characteristics		All Participants (*n* = 117)	CG (*n* = 56)	EG (*n* = 63)	*p*-Value
Age: mean ± SD (years)		45.65 ± 8.86	46.89 ± 6.59	44.54 ± 10.40	0.149
Weight: mean ± SD (Kg)		63.59 ± 10.59	64.62 ± 10.04	62.67 ± 11.05	0.318
Height: mean ± SD (cm)		162.56 ± 5.95	163.45 ± 5.83	161.78 ± 5.99	0.128
BMI: mean ± SD (kg/m^2^)		24.03 ± 3,63	24.15 ± 3.31	23.93 ± 3.92	0.742
Nº pregnant: mean ± SD		1.54 ± 1.07	1.71 ± 1.06	1.38 ± 1.07	0.090
Nº delivery: mean ± SD		1.47 ± 1.05	1.63 ± 1.04	1.33 ± 1.05	0.130
Delivery type: *n* (%)	None	28 (23.5)	10 (35.7)	18 (64.3)	0.533 **
	Vaginal	68 (57.1)	34 (50)	34 (50)	
	Caesarean	12 (10.1)	7 (58.3)	5 (41.7)	
	Both	10 (8.4)	5 (45.5)	6 (54.5)	
Smoking: *n* (%)	No	100 (84)	49 (49)	51 (51)	0.33
	Yes	19 (16)	7 (36.8)	12 (63.2)	
Exercise: *n* (%)	No	56 (47.1)	27 (48.2)	29 (51.8)	0.812
	yes	63 (52.9)	29 (46)	34 (54)	
RMSX-OE: mean ± SD (mm)		0.59 ± 0.11	0.58 ± 0.12	0.59 ± 0.10	0.833
RMSX-CE: mean ± SD(mm)		0.64 ± 0.12	0.64 ± 0.11	0.64 ± 0.13	0.962
RMSY-OE: mean ± SD (mm)		0.42 ± 0.99	0.41 ± 0.10	0.43 ± 0.09	0.354
RMSY-CE: mean ± SD (mm)		0.50 ± 0.15	0.49 ± 0.14	0.50 ± 0.16	0.796
V-OE: mean ± SD (mm/s)		15.97 ± 3.57	15.95 ± 4.31	15.98 ± 2.80	0.964
V-EC: mean ± SD (mm/s)		16.78 ± 3.20	16.56 ± 2.79	16.98 ± 3.53	0.476
S-O: mean ± SD (mm^2^)		42.56 ± 41.34	35.69 ± 31.22	48.67 ± 48.03	0.087
S-CE: mean ± SD (mm^2^)		85.76 ± 137.31	77.32 ± 94.72	93.25 ± 166.74	0.530
TrA relaxation: mean ± SD		0.40 ± 0.08	0.40 ± 0.09	0.39 ± 0.08	0.921
TrA contraction: mean ± SD		0.70 ± 0.14	0.74 ± 0.14	0.67 ± 0.13	0.239

1. Notes: Data collected as the means; ± standard deviations and; frequencies (percentages) for continuous and categorical variables respectively; CG: Control Group; EG: Experimental Group; SD: Standard Deviation; kg: kilograms; cm: centimeters; mm: millimeters; s: second; IBM: Body Mass Index; Nº: number; RMSX: mediolateral mean displacements of the center of pressure; RMSY: anteroposterior means displacements of the center of pressure; V: velocity of the center of pressure movements; S: area of the center of pressure movement. OE: open eyes; CE: closed eyes. 2. ** Fisher’s exact test.

**Table 3:** Between-group and within-group comparison of the stabilometric parameters and TrA measures.

	Control Group (*n* = 56)		Experimental Group (*n* = 63)		Group			Time			Group × Time		
	Pre	Post	Pre	Post	F (1.117)	*p*-Value	η^2^	F (1.117)	*p*-Value	η^2^	F (1.117)	*p*-Value	η^2^
RMSX-OE (mm)	0.58 ± 0.12	0.59 ± 0.11	0.59 ± 0.10	0.58 ± 0.10	0.086	0.770	0.001	0.025	0.875	0.001	0.411	0.52	0.003
RMSX-CE (mm)	0.64 ± 0.11	0.67 ± 0.14	0.64 ± 0.13	0.63 ± 0.10	1.088	0.299	0.009	1.153	0.285	0.010	2.714	0.102	0.023
RMSY-OE (mm)	0.41 ± 0.10	0.40 ± 0.10	0.43 ± 0.09	0.38 ± 0.09	0.032	0.859	0.001	12.585	0.001*	0.097	5.095	0.026*	0.042
RMSY-CE (mm)	0.49 ± 0.14	0.45 ± 0.12	0.50 ± 0.16	0.42 ± 0.10	0.292	0.590	0.002	17.049	0.001*	0.127	1.315	0.254	0.011
V-OE (mm/s)	15.95 ± 4.31	17.00 ± 2.66	15.98 ± 2.80	15.21 ± 2.99	3.217	0.075	0.027	0.182	0.670	0.002	7.401	0.008**	0.059
V-CE (mm/s)	16.56 ± 2.79	16.97 ± 2.66	16.98 ± 3.53	15.75 ± 2.78	0.761	0.385	0.006	1.861	0.175	0.016	7.648	0.007*	0.061
S-OE (mm^2^)	35.69 ± 31.22	23.14 ± 18.59	48.67 ± 48.03	15.16 ± 2.55	0.369	0.545	0.003	37.168	0.001*	0.241	7.705	0.006**	0.062
S-CE (mm^2^)	77.32 ± 94.72	53.71 ± 52.75	93.25 ± 166.74	50.19 ± 50.62	0.158	0.691	0.001	9.151	0.003*	0.073	0.779	0.379	0.007
TrA relaxation	0.40 ± 0.09	0.41 ± 0.09	0.39 ± 0.08	0.40 ± 0.08	0.013	0.911	0.001	0.904	0.344	0.008	0.001	0.989	0.001
Tra contraction	0.74 ± 0.14	0.74 ± 0.17	0.67 ± 0.13	0.75 ± 0.17	1.835	0.178	0.015	15.44	0.001*	0.117	13.057	0.001**	0.100

*p* value < 0.05 Post vs. Pre intervention; ** *p* value < 0.05 Intervention vs. Control; Notes: Data collected as means, standard deviations, frequencies, and percentages for continuous and categorical variables. CG: Control Group; EG: Experimental Group; η^2^: Eta Squared; RMSX: mediolateral mean displacements of the center of pressure; RMSY: anteroposterior mean displacements of the center of pressure; V: velocity of the center of pressure movements; S: area of the center of pressure movement. OE: Open eyes; CE: Closed eyes.
